# Minimally invasive or conventional sternotomy for concomitant mitral valve surgery and surgical ablation for atrial fibrillation: propensity score study

**DOI:** 10.3389/fcvm.2026.1821864

**Published:** 2026-07-28

**Authors:** Robert Kashapov, Sergey Khrushchev, Ravil Sharifulin, Bashir Tsaroev, Murtazali Murtazaliev, Mirzo Mansurov, Anton Zalesov, Igor Demin, Alexander Bogachev-Prokophiev

**Affiliations:** 1 E. Meshalkin National Medical Research Center, Institute of Cardiovascular Pathology Research, Novosibirsk, Russia; 2 Siberian Branch of the Russian Academy of Sciences, Sobolev Institute of Mathematics, Novosibirsk, Russia

**Keywords:** atrial fibrillation, Cox-Maze procedure, minimally invasive surgery, mitral valve, propensity score study

## Abstract

**Background:**

Surgical ablation for atrial fibrillation (AF) during mitral valve surgery is performed via minimally invasive (MICS) or conventional sternotomy (CS) approaches, with a paucity of comparative data.

**Objective:**

This study compared the efficacy and safety of MICS vs. CS for concomitant mitral valve surgery and AF ablation.

**Methods:**

From January 2012 to December 2024, 496 combined procedures were performed. After exclusions, 457 patients were analyzed (73 MICS, 384 CS). Propensity score matching (PSM) was used to create balanced groups for comparison.

**Results:**

After PSM (61 MICS, 191 CS). cardiopulmonary bypass time, aortic cross-clamp time, and total operative duration were longer in the MICS group (*p* < 0.001). The MICS group also had a longer ICU stay [2 (1–3) vs. 1 (1–2), *p* < 0.001] and greater postoperative drainage (MICS—IQR 350–750 mL vs. CS—IQR 250–500 mL, *p* < 0.001). No significant differences were observed in the incidence of major adverse cardiac and cerebrovascular events (MACE) or mortality. At discharge, more MICS patients were in sinus rhythm (95% vs. 70%, *p* = 0.003). During long-term follow-up (mean 3.15 years), there were no significant differences in overall survival (HR = 0.69, 95% CI: 0.22–2.21, *p* = 0.53), freedom from atrial tachyarrhythmias (HR = 0.51, 95% CI: 0.25–1.05, *p* = 0.068), or freedom from permanent pacemaker implantation [HR = 1.13 (0.20–6.49), *p* = 0.89].

**Conclusion:**

Despite longer perfusion times and certain early recovery metrics, the MICS for combined mitral valve and AF surgery offers comparable long-term safety, efficacy, and rhythm control outcomes to CS. MICS should be considered a viable alternative, contingent upon surgical expertise and individualized patient assessment.

## 1 Introduction

Atrial fibrillation (AF) is the most prevalent sustained cardiac arrhythmia in clinical practice (1). In mitral valve disease, AF often emerges as a pathophysiological consequence of disease progression, further impairing patients' quality of life. Surgical strategies for concomitant mitral valve and AF surgery have evolved substantially, making the minimally invasive approach a conventional option for these combined procedures.

However, minimally invasive access can limit surgical visualization, potentially affecting the precise placement of ablation devices. Suboptimal exposure may result in discontinuous ablation lines, reducing procedural efficacy. Furthermore, to shorten cardiopulmonary bypass (CPB) and aortic cross-clamp (ACC) times, surgeons might perform a left atrial-only lesion set instead of a full bi-atrial set, potentially increasing the risk of early postoperative arrhythmia recurrence. This study aimed to perform a comparative analysis of perioperative outcomes and long-term efficacy in patients undergoing combined mitral valve and AF surgery via either a minimally invasive or a conventional sternotomy approach.

## 2 Materials and methods

**Figure 1:**
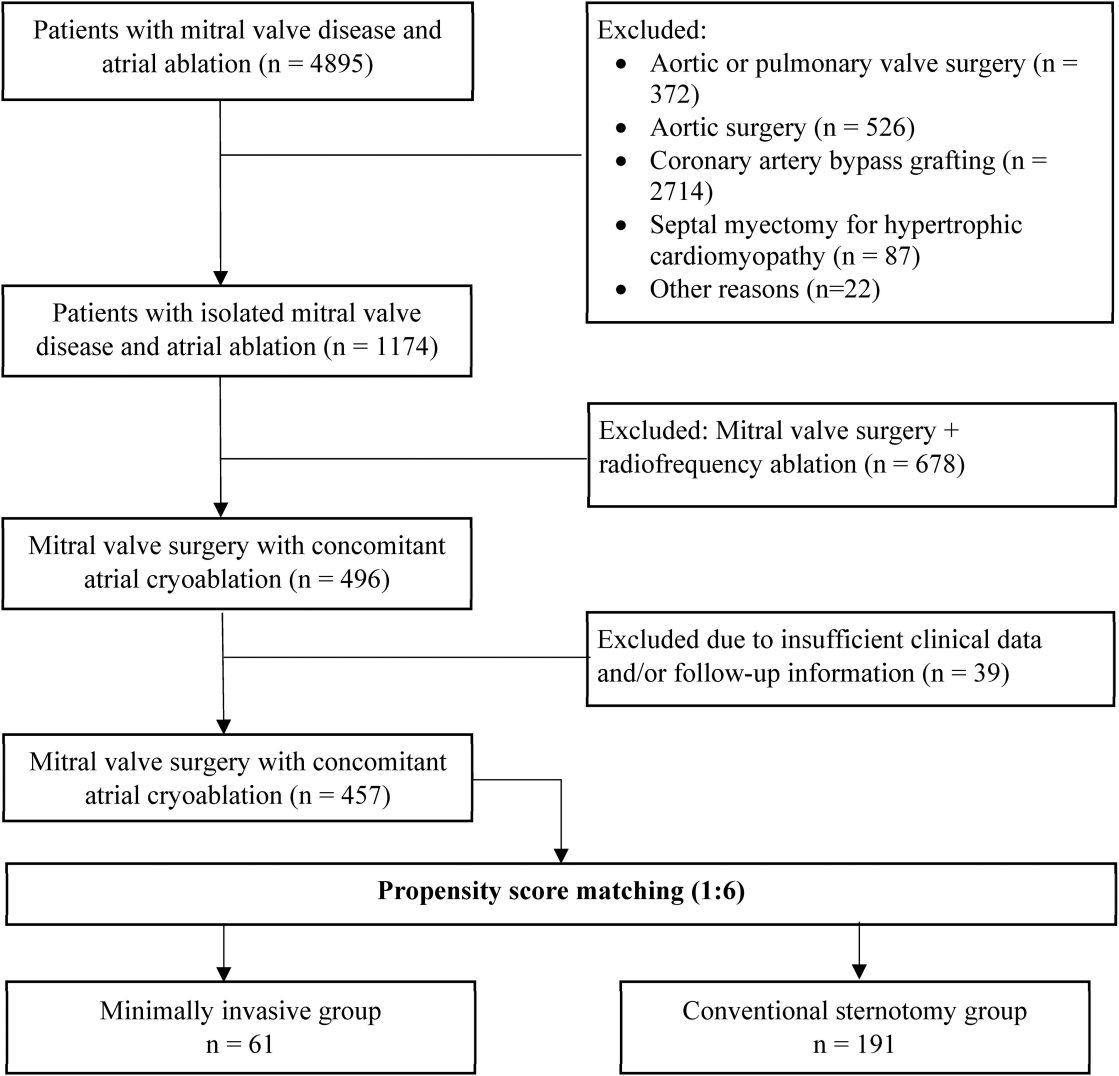
Study flow diagram.

A retrospective cohort analysis was conducted using the electronic medical records database of the National Medical Research Center named after E.N. Meshalkin. The study period was from January 2012 to December 2024. All patients included in the study had definitive indications for mitral valve surgery and presented with documented AF of varying clinical forms. The exclusion criteria were defined as follows: the need for any concomitant cardiac surgery other than combined procedures on the tricuspid valve, a history of previous cardiac surgery, prior percutaneous catheter-based ablation, and the use of radiofrequency ablation devices during the index procedure. A total of 496 patients who underwent concomitant mitral valve surgery and cryoablation for atrial fibrillation were identified. Thirty-nine patients were excluded because of incomplete data on the covariates included in the statistical analysis and/or insufficient follow-up data on the study endpoints. The final analysis cohort comprised 457 patients (Figure 1). All included patients were deemed equally suitable, from both anatomical and clinical standpoints, for either a minimally invasive or a standard surgical approach. Accordingly, in the conceptual framework of this study, the primary determinant of access selection was the operating surgeon's individual preference and the patient's personal preference. Within this cohort, 73 procedures were performed via a right mini-thoracotomy approach [minimally invasive cardiac surgery (MICS) group], while 384 patients were operated on via a conventional sternotomy (CS group).

The study protocol was reviewed and approved by the local Institutional Ethics Committee of the E.N. Meshalkin National Medical Research Center (№ 3–82). Given the retrospective design of the study utilizing anonymized clinical data, the requirement for obtaining individual patient informed consent was waived by the institutional ethics committee.

### 2.1 Surgical technique and postoperative management

A key methodological distinction between the groups, beyond the surgical access, pertained to the strategy for establishing cardiopulmonary bypass. In the MICS group, CPB was instituted via femoral arterial and venous cannulation. In cases of insufficient venous drainage, the circuit was supplemented by additional cannulation of the internal jugular vein. Conversely, in the CS group, CPB was established through central cannulation of the great vessels. Following the initiation of aortic cross-clamping and cardioplegic arrest, access to the mitral valve was attained via a dissection plane posterior to the interatrial groove.

For the ablation procedure, the AtriCure cryoICE cryoprobe (AtriCure, Inc., Mason, OH, USA) was employed, which utilizes nitrous oxide as its cryogen. The ablation protocol consisted of applying a temperature of −70°C for a duration of two minutes per lesion. The lesion set was identical in both surgical groups. The left atrial lesion set comprised pulmonary vein isolation with subsequent left atrial wall ablation, incorporating additional connecting lines to the mitral valve annulus, the left atrial appendage, and the coronary sinus. The right atrial lesion set consisted of four distinct lines: to both caval veins, a line to the right atrial appendage, and an ablation line to the tricuspid valve annulus. The technique for left atrial appendage (LAA) occlusion in the CS group was performed according to the operating surgeon's preference. In the minimally invasive group, LAA occlusion prior to 2022 was performed using a double-row endocardial suture technique. Since 2022, LAA exclusion has been routinely achieved with an ENDO GIA™ Universal Auto Suture™ stapling device (Medtronic, Minneapolis, MN, USA).

Surgical management of the mitral valve was patient-specific, with the choice between repair and replacement based on intraoperative assessment. Procedural success was confirmed by intraoperative transesophageal echocardiography.

Patients who received a mechanical prosthesis were prescribed vitamin K antagonist (VKA, warfarin) anticoagulation therapy with a target INR of 3.0 (range 2.5–3.5). For patients undergoing mitral valve replacement with a biological prosthesis or mitral valve repair, anticoagulation therapy with a VKA (target INR 2.5, range 2.0–3.0) was administered for the first 3 months. After this period, a reassessment of cardiac rhythm and the CHA₂DS₂-VASc score was performed, and anticoagulation therapy was adjusted accordingly.

Antiarrhythmic Management: For the prevention of early postoperative atrial tachyarrhythmias, amiodarone (intravenous load followed by oral, e.g., 200 mg daily) or a beta-blocker (e.g., metoprolol) was prescribed for the first 6–12 weeks. In cases of arrhythmia recurrence, the first-line strategy was pharmacological cardioversion with an amiodarone loading regimen. If ineffective, electrical cardioversion was performed.

### 2.2 Endpoints and follow-up

The primary endpoint was freedom from atrial tachyarrhythmias, evaluated both in the early (≤30 days) and late (>30 days) postoperative periods. An endpoint event was defined as any recurrence of atrial arrhythmia (including atrial fibrillation, atrial flutter, or atrial tachycardia) lasting more than 30 s, as documented by objective cardiac rhythm monitoring. During the hospitalization, rhythm assessment was conducted using continuous cardio-telemetry, standard 12-lead electrocardiography, and 24 h Holter monitoring. Following discharge, rhythm assessment was primarily performed using 24 h Holter monitoring.

Secondary endpoints comprised a comparative analysis of overall survival between the two groups and the rate of permanent pacemaker implantation. Furthermore, adverse events were evaluated in compliance with the established guidelines for reporting mortality and morbidity after cardiac valve interventions (2).

Postoperative outcomes were stratified into early events (occurring during the index hospital stay) and late events (occurring after hospital discharge). Long-term follow-up after discharge was conducted through either in-person visits to the medical institution or via structured telephone interviews. Patient outcomes were assessed by requesting and reviewing medical documentation sent by mail.

### 2.3 Statistical analysis

To mitigate the substantial baseline imbalance between the conventional sternotomy (CS, *n* = 384) and minimally invasive (MICS, *n* = 73) groups, a propensity score matching (PSM) analysis was performed. Given the significant disparity in group sizes, a 1:6 matching protocol was employed, allowing each patient from the smaller MICS group to be matched with no more than six patients from the CS group.

This ratio was chosen to maximize the effective sample size from the large CS pool. The final achieved matching ratio was approximately 3:1 (191 CS to 61 MICS).

The propensity score was estimated using a multivariable logistic regression model with the surgical approach (MICS vs. CS) as the dependent variable. Variables for the model were selected *a priori* based on clinical expertise and published evidence. Nearest neighbor matching without replacement was performed using a caliper of 0.1 for the raw (untransformed) propensity scores. This caliper value was selected based on established recommendations to balance bias reduction and sample size preservation.

**Figure 2:**
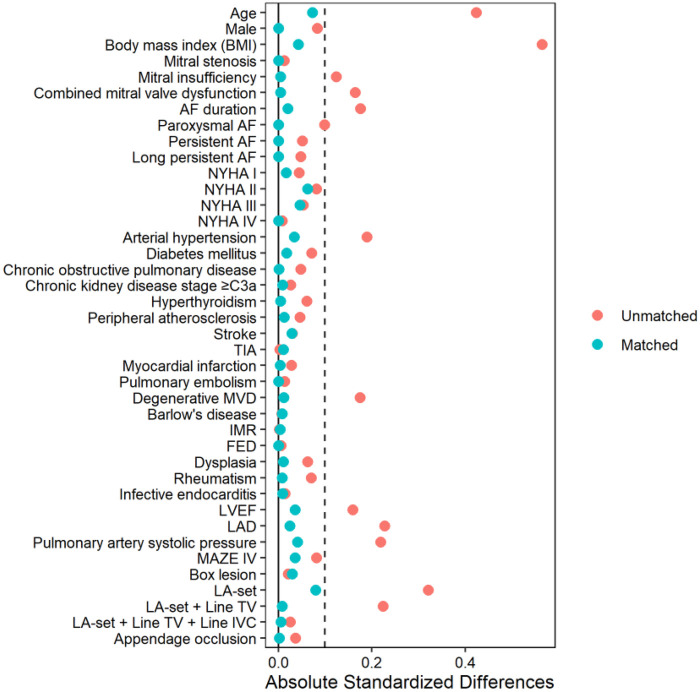
Standardized mean differences for patient characteristics between unmatched and matched cohorts. AF, atrial fibrillation; NYHA, New York heart association; TIA, transient ischemic attack; MVD, mitral valve disease; IMR, ischemic mitral regurgitation; FED, fibroelastic deficiency; LVEF, left ventricular ejection fraction; LAD, left atrial diameter; LA, left atrium; TV, tricuspid valve; IVC, inferior vena cava.

The balance of covariates before and after matching was assessed using absolute standardized differences (ASD). The ASD was calculated as the absolute standardized difference between means for continuous variables and as the absolute difference between proportions for binary variables. In addition, variance ratios were calculated for continuous variables. An ASD < 0.1 and variance ratios between 0.5 and 2.0 for continuous variables were considered indicative of adequate balance between the matched groups. Following matching, the cohort comprised 252 patients: 61 in the MICS group and 191 in the CS group. All included covariates were well-balanced in the matched sample (Figure 2). Analysis of the matched cohort was performed using weighting, where each matched CS patient was assigned, a weight corresponding to the inverse of the number of CS patients matched to a given MICS patient.

Missing data were handled using complete-case analysis, as the proportion of missing values for any variable was negligible.

Continuous variables are presented as median (interquartile range, IQR). For unmatched groups, comparisons of continuous variables were performed using the Mann–Whitney *U*-test. For the matched groups, comparisons of continuous variables accounted for the matched structure using quantile regression. Categorical variables are presented as counts (percentages). For unmatched data, they were compared using the chi-squared test or Fisher's exact test, as appropriate. For matched data, categorical variables accounted for the matched structure using conditional logistic regression.

The primary endpoint, freedom from atrial tachyarrhythmias, and secondary time-to-event endpoints (overall survival, freedom from pacemaker implantation) were analyzed using the Kaplan–Meier method within the matched cohort. Univariable Cox proportional hazards regression was used to estimate hazard ratios (HR) with 95% confidence intervals (CI). The proportional hazards assumption for the Cox regression models was tested using Schoenfeld residuals. In all cases, the proportional hazards assumption was satisfied (global test *p* > 0.05 for all models).

Statistical significance was set at *p* < 0.05. Statistical calculations were performed using R version 4.3.3 (R Core Team, 2018; R: A language and environment for statistical computing. R Foundation for Statistical Computing, Vienna, Austria, URL: https://www.R-project.org/).

## 3 Results

### 3.1 Baseline preoperative data

The baseline characteristics of the matched and unmatched cohorts are presented in Table 1. Prior to propensity score matching (PSM), the groups demonstrated significant differences, most notably in the variables of age and body mass index. The final matched cohort comprised 252 patients 191 patients in the CS group and 61 patients in the MICS group.

**Table 1:** Baseline characteristics.

Variable	Unmatched cohort (*n* = 457)			Matched cohort (*n* = 252)		
	CS (*n* = 384)	MICS (*n* = 73)	ASD	CS (*n* = 191)	MICS (*n* = 61)	ASD
Age	59 ± 9	55 ± 8	0.424	57 ± 10	55 ± 8	0.073
Male	152 (40%)	35 (48%)	0.084	87 (46%)	29 (48%)	0.001
BMI	29.0 ± 6.4	25.9 ± 4.4	0.564	27.2 ± 4.6	26.3 ± 4.6	0.043
AH	236 (61%)	31 (42%)	0.19	102 (53%)	27 (44%)	0.034
DM	38 (9.9%)	2 (2.7%)	0.072	16 (8.4%)	2 (3.3%)	0.018
COPD	29 (7.6%)	2 (2.7%)	0.048	9 (4.7%)	2 (3.3%)	0.001
CKD > C3a	26 (6.8%)	3 (4.1%)	0.027	12 (6.3%)	3 (4.9%)	0.009
Hyperthyroidism	76 (20%)	10 (14%)	0.061	34 (18%)	9 (15%)	0.004
Peripheral atherosclerosis	44 (11%)	5 (6.8%)	0.046	17 (8.9%)	4 (6.6%)	0.013
Stroke	36 (9.4%)	9 (12%)	0.03	20 (10%)	6 (9.8%)	0.029
TIA	4 (1.0%)	1 (1.4%)	0.003	3 (1.6%)	1 (1.6%)	0.011
MI	16 (4.2%)	1 (1.4%)	0.028	4 (2.1%)	1 (1.6%)	0.004
Pulmonary embolism	5 (1.3%)	0 (0%)	0.013	0 (0%)	0 (0%)	0
Type of AF						
Paroxysmal AF	104 (27%)	27 (37%)	0.099	66 (35%)	24 (39%)	0
Persistent AF	88 (23%)	13 (18%)	0.051	41 (21%)	12 (20%)	0
Long pers. AF	192 (50%)	33 (45%)	0.048	84 (44%)	25 (41%)	0
NYHA functional class						
NYHA I	4 (1.0%)	4 (5.5%)	0.044	2 (1.0%)	3 (4.9%)	0.016
NYHA II	147 (38%)	22 (30%)	0.081	69 (36%)	20 (33%)	0.063
NYHA III	227 (59%)	47 (64%)	0.053	120 (63%)	38 (62%)	0.046
NYHA IV	3 (0.8%)	0 (0%)	0.008	0 (0%)	0 (0%)	0
Mitral valve dysfunction						
Stenosis	53 (14%)	11 (15%)	0.013	31 (16%)	10 (16%)	0
Insufficiency	189 (49%)	45 (62%)	0.124	103 (54%)	37 (61%)	0.005
Combined	142 (37%)	15 (21%)	0.164	55 (29%)	13 (21%)	0.005
Etiology of Mitral Valve Dysfunction						
Degenerative	75 (20%)	27 (37%)	0.175	50 (26%)	21 (34%)	0.011
Barlow	8 (2.1%)	1 (1.4%)	0.007	3 (1.6%)	1 (1.6%)	0.008
IMR	6 (1.6%)	1 (1.4%)	0.002	4 (2.1%)	1 (1.6%)	0.004
FED	2 (0.5%)	0 (0%)	0.005	0 (0%)	0 (0%)	0
Dysplasia	66 (17%)	8 (11%)	0.062	27 (14%)	8 (13%)	0.011
Rheumatism	206 (54%)	34 (47%)	0.071	99 (52%)	28 (46%)	0.008
Endocarditis	16 (4.2%)	2 (2.7%)	0.014	8 (4.2%)	2 (3.3%)	0.009
AF duration	3.2 ± 4.5	4.1 ± 5.6	0.176	3.5 ± 5.2	3.4 ± 3.9	0.02
LVEF (%)	59 ± 9	60 ± 9	0.16	60 ± 7	60 ± 9	0.036
LAD (mm)	6.66 ± 0.76	6.48 ± 0.83	0.227	6.57 ± 0.81	6.55 ± 0.83	0.025
PASP	47 ± 11	44 ± 10	0.219	47 ± 11	45 ± 10	0.041
Ablation Strategies						
MAZE IV	126 (33%)	18 (25%)	0.082	57 (30%)	14 (23%)	0.036
Box Lesion	13 (3.4%)	4 (5.5%)	0.021	11 (5.8%)	3 (4.9%)	0.03
LA-set	124 (32%)	47 (64%)	0.321	97 (51%)	40 (66%)	0.08
LA-set + Line TV	102 (27%)	3 (4.1%)	0.225	20 (10%)	3 (4.9%)	0.008
LA-set + Line TV + Line IVC	15 (3.9%)	1 (1.4%)	0.025	6 (3.1%)	1 (1.6%)	0.005
Appendage occlusion	293 (76%)	53 (73%)	0.037	143 (75%)	47 (77%)	0.002

AF, atrial fibrillation; AH, arterial hypertension; BMI, body mass index; CKD, chronic kidney disease; COPD, chronic obstructive pulmonary disease; CS, conventional sternotomy; DM, diabetes mellitus; FED, fibroelastic deficiency; IMR, ischemic mitral regurgitation; LAD, diameter of the left atrium; LA-set, left atrial ablation set; Line IVC, ablation line to inferior vena cava; Line TV, ablation line to tricuspid valve; LVEF, left ventricular ejection fraction (before operation); MI, myocardial infarction; MICS, minimally invasive cardiac surgery; NYHA, New York heart association; PASP, pulmonary artery systolic pressure; TIA, transient ischemic attack.

### 3.2 Operative data

Intraoperative data are summarized in Table 2. The most anticipated significant differences were observed in the duration of cardiopulmonary bypass (CPB) and aortic cross-clamp (ACC) time. In the MICS group, the duration of CPB, ACC, and the total operative time were significantly longer compared to the CS group (*p* < 0.001 for all). A significantly greater number of concomitant mitral valve repair procedures were performed via minimally invasive approach compared to the conventional sternotomy (35% vs. 54%, *p* = 0.033). However, the number of tricuspid valve repair procedures was higher in the standard access group (57% vs. 20%, *p* < 0.001).

**Table 2:** Operative data.

Variable	Unmatched cohort (*n* = 457)			Matched cohort (*n* = 252)		
	CS (*n* = 384)	MICS (*n* = 73)	*p*—value	CS (*n* = 191)	MICS (*n* = 61)	*p*—value
Duration of operation	200 (180–240)	295 (270–340)	<0.001	200 (180–240)	295 (270–340)	<0.001
CPB time (min)	101 (89–120)	186 (159–224)	<0.001	101 (89–120)	186 (159–224)	<0.001
ACC time (min)	78 (66–89)	115 (99–135)	<0.001	78 (66–89)	115 (99–135)	<0.001
Re-ACC	4 (1.0%)	3 (4.1%)	0.085	2 (1.0%) [0.5%]*	3 (4.9%)	0.054
MV replacement	256 (67%)	32 (44%)	<0.001	120 (63%) [57.1%]	27 (44%)	0.032
MV repair	121 (32%)	40 (55%)	<0.001	66 (35%) [41.4%]	33 (54%)	0.033
TV repair	219 (57%)	14 (19%)	<0.001	109 (57%) [55.5%]	12 (20%)	<0.001
TV replacement	8 (2.1%)	3 (4.1%)	0.39	5 (2.6%) [1.8%]	3 (4.9%)	0.26

ACC, aortic cross clamp; CPB, cardiopulmonary bypass time; CS, conventional sternotomy; MICS, minimally invasive cardiac surgery; MV, mitral valve; re-ACC, re-occlusion of the aorta; TV, tricuspid valve.

* For the matched cohort, percentages in bold brackets [%] represent weighted percentages accounting for the 1:k matching structure.

**Figure 3:**
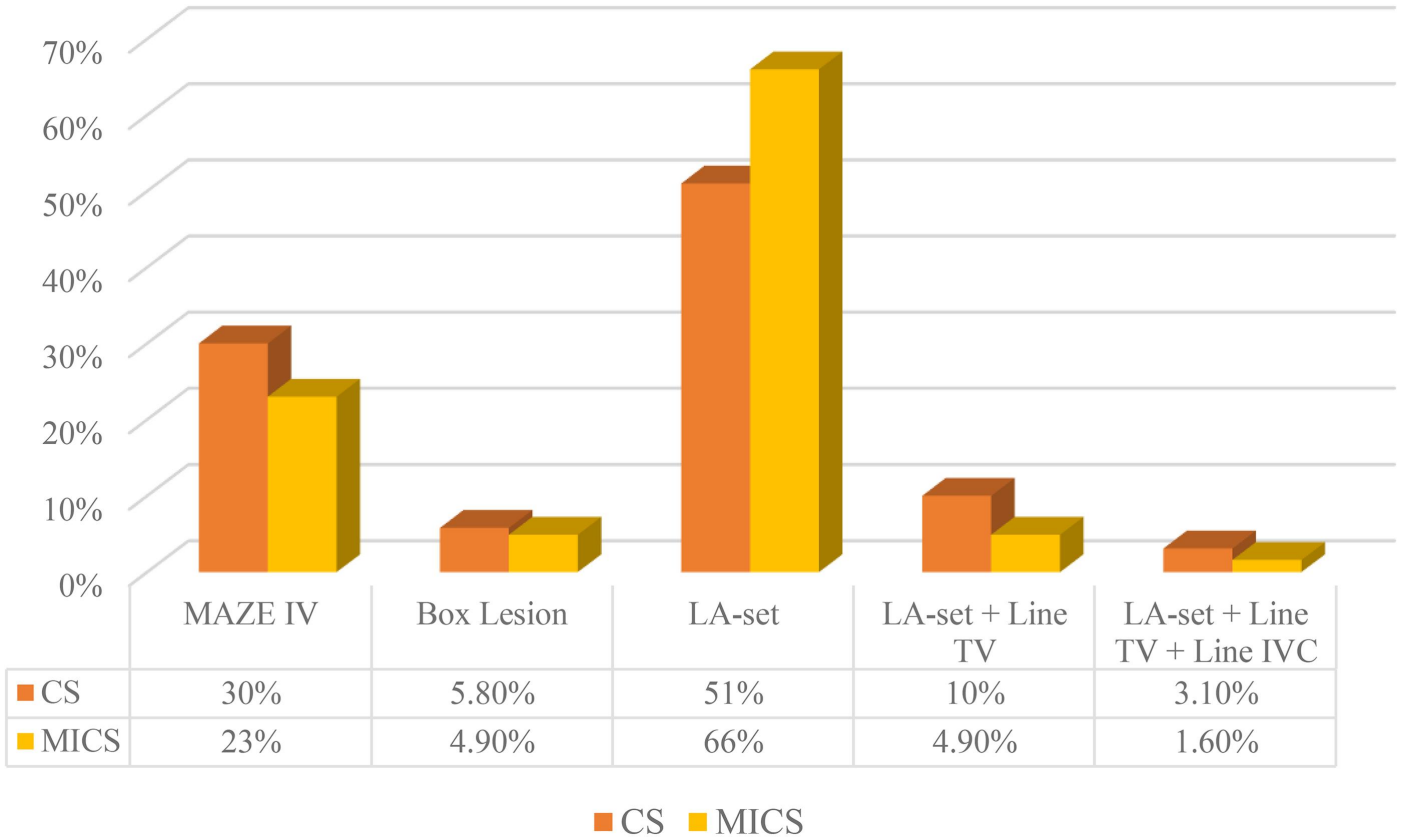
Ablation strategies after propensity score matching. CS, conventional sternotomy; LA-set, left atrial ablation set; Line IVC, ablation line to inferior vena cava; Line TV, ablation line to tricuspid valve; MICS, minimally invasive cardiac surgery.

Following propensity score matching, all ablation strategies were well-balanced (Table 1). The choice of surgical ablation lesion set was determined based on the type and duration of atrial fibrillation, as well as surgeon preference. A graphical representation of the employed ablation lesion sets is provided in Figure 3.

### 3.3 Early postoperative outcomes

Results for hospital outcomes and early postoperative data are presented in Table 3. The analysis was performed using the matched dataset. During the hospitalization period, the groups demonstrated no significant differences in the incidence of major adverse cardiovascular events (MACE), with the proportion of all events being approximately equal. Furthermore, no differences were observed between the groups regarding the duration of inotropic support and mechanical ventilation.

**Table 3:** Early postoperative data.

Variable	Unmatched cohort (*n* = 457)			Matched cohort (*n* = 252)		
	CS (*n* = 384)	MICS (*n* = 73)	*p*—value	CS (*n* = 191)	MICS (*n* = 61)	*p*—value
ICU stay (days)	1 (1–2)	2 (1–3)	0.005	1 (1–2)	2 (1–3)	<0.001
LOS (d)	14 (11–18)	13 (11–21)	0.76	14 (11–19)	14 (11–21)	>0.99
Inotropic support (h)	8 (0–19)	12 (2–30)	0.02	7 (0–18)	10 (1–23)	0.062
Mechanical Ventilation (h)	6.0 (4.0–10.0)	7.0 (5.0–12.0)	0.34	6 (4–9)	7 (5–11)	0.074
Bleeding (mL)	375 (288–500)	500 (350–750)	<0.001	350 (250–500)	500 (350–750)	<0.001
Revision due to bleeding	18 (4.7%)	6 (8.2%)	0.25	6 (3.1%) [2.5%]*	6 (9.8%)	0.068
Operative mortality	0 (0%)	0 (0%)	—	0 (0%) [0%]	0 (0%)	—
Pneumonia	7 (1.8%)	4 (5.5%)	0.082	3 (1.6%) [1.9%]	3 (4.9%)	0.18
Redo cardiac surgery*	2 (0.5%)	1 (1.4%)	0.41	1 (0.5%) [0.8%]	1 (1.6%)	0.53
MI	3 (0.8%)	2 (2.7%)	0.18	1 (0.5%) [0.8%]	1 (1.6%)	0.81
Stroke	1 (0.3%)	2 (2.7%)	0.068	1 (0.5%) [0.8%]	1 (1.6%)	0.81
TIA	2 (0.5%)	0 (0%)	>0.99	2 (1.0%) [0%]	0 (0%)	>0.99
Thromboembolism	0 (0%)	0 (0%)	—	0 (0%) [0%]	0 (0%)	—

CS, conventional sternotomy; ICU, intensive care unit; LOS, length of stay; MICS, minimally invasive cardiac surgery; MI, myocardial infarction; TIA, transient ischemic attacks. * Redo cardiac surgery—repeated cardiac surgery during a single hospitalization.

* For the matched cohort, percentages in bold brackets [%] represent weighted percentages accounting for the 1:k matching structure.

Patients in the CS group had a significantly shorter ICU stay [1 (1–2) vs. 2 (1–3), *p* < 0.001] compared to the control group, with no significant difference in overall hospital length of stay (*p* > 0.99). Despite substantial data variability in the MICS group (IQR 350–750 mL vs. IQR 250–500 mL in the CS group), the matched analysis revealed that it had a significantly higher volume of postoperative drainage blood loss (*p* < 0.001). One reoperation was reported in each group, both attributable to recurrent mitral regurgitation post-repair, confirmed by transthoracic echocardiography. There were no cases of intraoperative or in-hospital mortality in either group.

### 3.4 Follow-up survival

**Figure 4:**
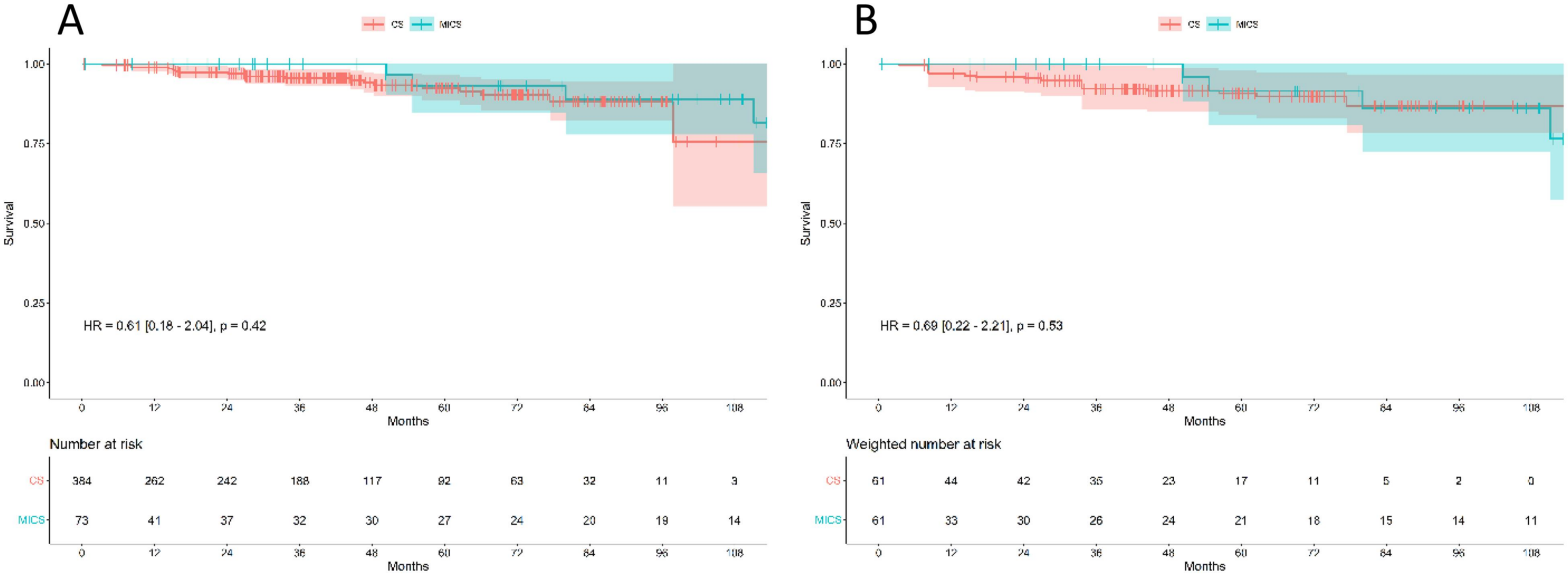
Kaplan–meier survival curve: all-cause mortality is shown for the unmatched **(A)** and matched **(B)** cohorts. CS, conventional sternotomy group; HR, hazard ratio; MICS, minimally invasive cardiac surgery group.

**Figure 5:**
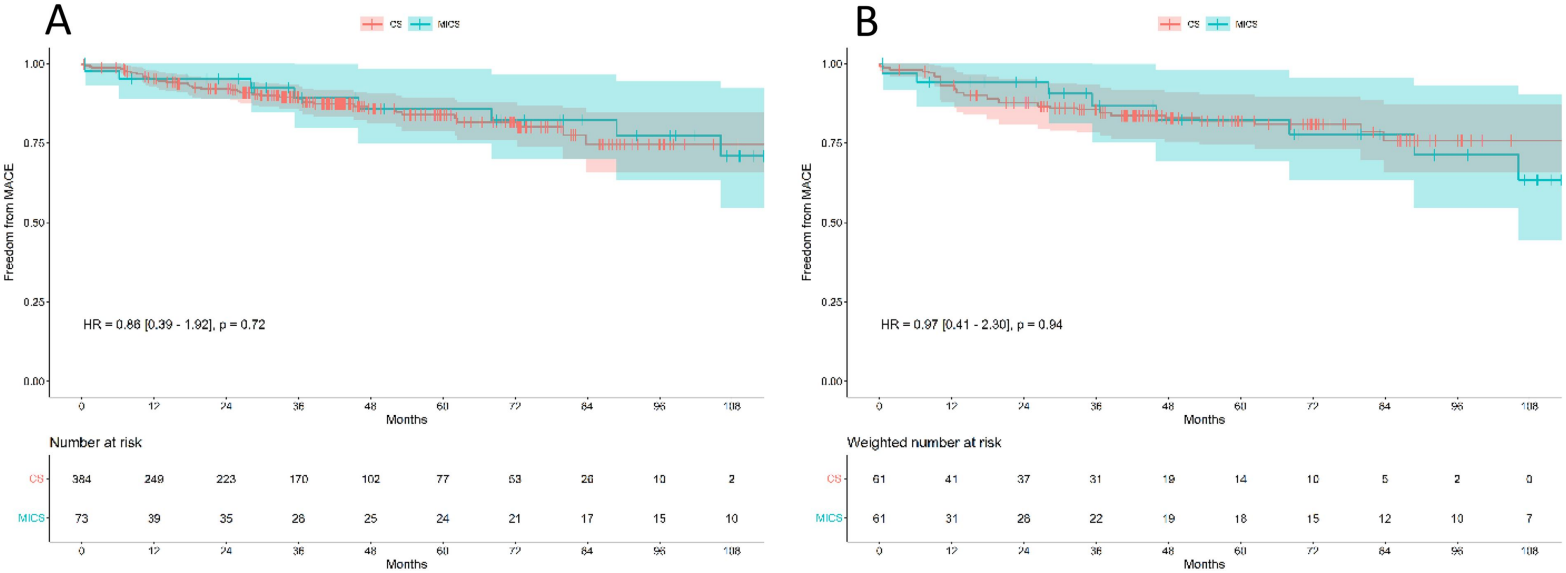
Major adverse cardiovascular event (MACE)-free survival for the unmatched **(A)** and matched **(B)** cohorts. CS, conventional sternotomy group; HR, hazard ratio; MICS, minimally invasive cardiac surgery group.

Follow-up was completed for 87% of patients, with a mean duration of 3.15 years for the entire cohort. Following PSM, Kaplan–Meier analysis for all-cause mortality showed no significant survival difference between the groups [HR = 0.69 (0.22–2.21), *p* = 0.53—matched cohort] (Figure 4B). Similarly, no difference was observed in the rate of MACE [HR = 0.97 (0.41–2.30), *p* = 0.94 matched cohort] (Figure 5B).

### 3.5 Rhythm outcomes

At the in-hospital stage, atrial arrhythmias occurred in 92 patients [72 patients (38%) in the CS group and 20 (33%) in the MICS group, *p* = 0.80]. In both groups, the method for rhythm restoration, depending on the duration and stability of the arrhythmia, involved either pharmacological or electrical cardioversion. During the early postoperative period, 14 patients required permanent pacemaker implantation [10 patients (5.2%) in the CS group and 4 patients (6.6%) in the MICS group, *p* = 0.87]. At the time of discharge, 192 out of 252 patients in the matched cohort were in sinus rhythm [134 (70%) patients in the CS group and 58 (95%) in the MICS group, *p* < 0.001].

**Figure 6:**
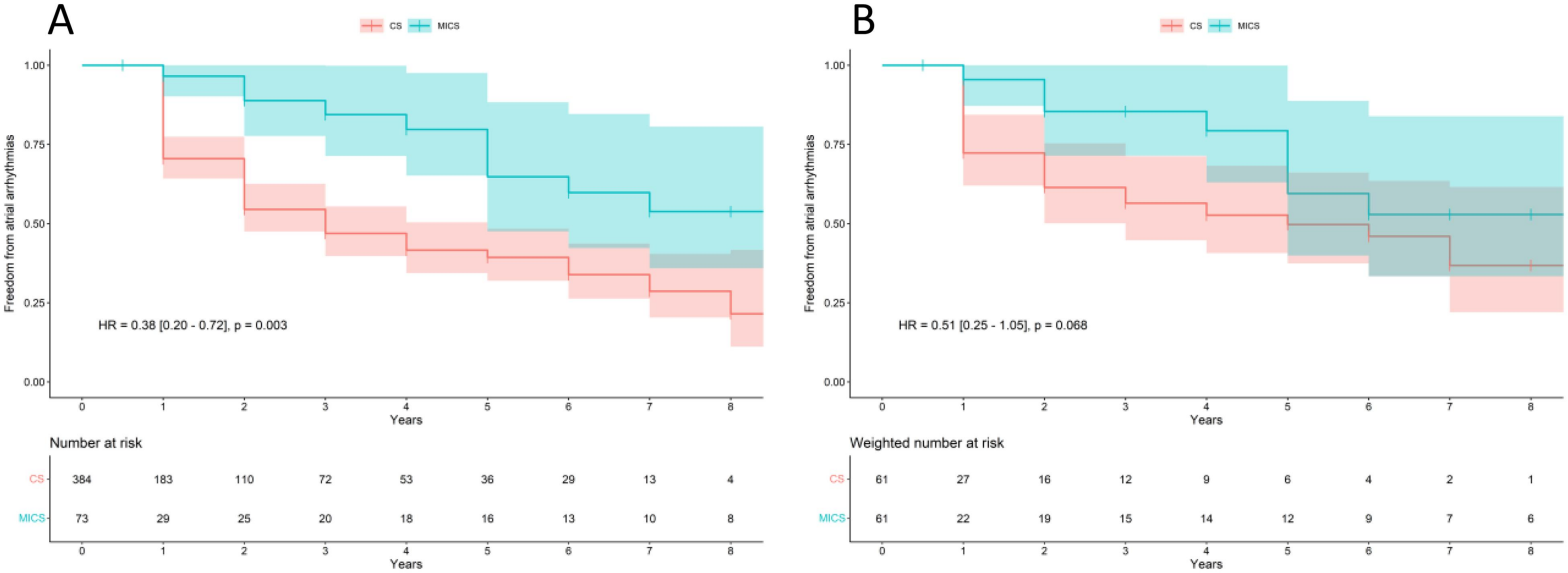
Freedom from atrial arrhythmia for the unmatched **(A)** and matched **(B)** cohorts. CS, conventional sternotomy group; HR, hazard ratio; MICS, minimally invasive cardiac surgery group.

The overlong follow-up analysis revealed no significant differences in freedom from atrial arrhythmia between the two patient groups [HR = 0.51 (0.25–1.05), *p* = 0.068] (Figure 6B). According to both univariable and multivariable Cox regression analysis (matched cohort), two factors were significantly associated with an increased risk of long-term arrhythmia recurrence: a longer preoperative arrhythmia duration [HR = 1.07 (95% CI 1.03, 1.11), *p* < 0.001] and early postoperative atrial fibrillation (EPOAF) recurrence (<30 days post-surgery) [HR = 3.13 (95% CI 1.55, 6.31), *p* = 0.001].

Analysis of arrhythmias requiring hospital readmission showed no significant differences between the groups [HR = 0.58 (0.18–1.85), *p* = 0.35] (Supplementary Figure S1B). Among the 252 patients (matched cohort), catheter ablation was performed in 2 patients to reduce the further progression of atrial fibrillation (one patient per group).

After discharge, all cases requiring permanent pacemaker implantation were analyzed. The freedom from pacemaker implantation rate was comparable between the groups [HR = 1.13 (0.20–6.49), *p* = 0.89] (Supplementary Figure S2B).

## 4 Discussion

The minimally invasive approach is often a valuable option for patients leading an active lifestyle. For this cohort, it is crucial not only to correct the cardiac defect but also to minimize the risk of adverse events in the postoperative period.

Among patients with mitral valve disease, the prevalence of concomitant AF is remarkably high, ranging from 30% to 84% (3–5). While AF alone increases the risk of ischemic stroke by 2.4 to 5-fold (6, 7), the presence of concomitant valvular heart disease further elevates this risk. Therefore, when introducing new surgical approaches, including minimally invasive techniques, it is imperative to assess their ability to provide durable long-term efficacy, particularly in terms of stroke prevention and arrhythmia control. The primary argument cited by clinicians against performing a combined mitral valve procedure and atrial ablation via a minimally invasive approach is the perceived reduced long-term efficacy in treating atrial fibrillation and the consequent increased risk of major adverse cardiovascular events. However, the validity of this assertion warrants critical examination.

Within the limited body of research in this specific area, the most recent and largest study to date is that by Yoon S et al. Over a 19-year period, the authors performed 360 combined mitral valve surgeries and surgical AF ablations via a right mini-thoracotomy (RMT group). The results were presented in a comparative analysis with a median sternotomy group [PSM analysis: matched cohort of 267 (sternotomy group) vs. 267 (RMT group)]. The authors found no statistically significant differences either in the rate of combined adverse events (HR: 0.76, *p* = 0.273) or in AF recurrence (HR: 0.72, *p* = 0.080) (8).

The particular clinical relevance of this question provided the impetus for our center to conduct a comparative systematic review, published in 2025 (9). Our meta-analysis, synthesizing data from 13 studies, further substantiates the equipoise between the two surgical approaches. It found no significant difference in freedom from atrial arrhythmias at one-year (*p* = 0.95) or two-year (*p* = 0.531) intervals. The analysis also found no differences in 30-day postoperative mortality between the groups (*p* = 0.709).

While our results also demonstrated no statistically significant differences in freedom from atrial tachyarrhythmias (*p* = 0.068), several noteworthy observations merit discussion. Contrary to the commonly reported data indicating a reduction in ICU length of stay (10, 11), the findings of the present study demonstrate an opposite trend. A detailed analysis of the database revealed that this correlation is attributable to isolated clinical cases in the minimally invasive group, whose complicated postoperative course led to a prolonged stay in the intensive care unit. Despite this, the overall length of hospital stay was comparable between the two groups (*p* > 0.99).

An unexpected finding was the significant difference in drainage blood loss (MICS group: IQR 350–750 mL vs. CS group: IQR 250–500 mL, *p* < 0.001). At the same time, no difference was observed in the rate of postoperative revision for bleeding (*p* = 0.068). In addressing this issue, it is important to consider not only the volume but also the nature of the drainage output. The development of exudative pericarditis/pleuritis represents a predictable autoimmune response following open cardiac surgical procedures (12, 13). A distinctive feature of the right thoracotomy approach is its more aggressive impact on the serous membranes of the thoracic cavity, particularly the parietal and visceral pleura, which can largely be avoided with a median sternotomy. This may serve as a potential contributing factor to the development of exudative pleuritis in the minimally invasive group and, consequently, to increased drainage loss.

Returning to the primary outcome (freedom from AA), two factors demonstrated a statistically significant impact on long-term arrhythmia recurrence, among which early postoperative atrial fibrillation is of particular interest. The pathogenesis of early postoperative arrhythmias is multifactorial, predominantly attributed to autonomic nervous system dysregulation, systemic inflammatory response, oxidative stress, and the arrhythmogenic potential of inotropic support (14). Consequently, the occurrence of EPOAF may not serve as a reliable surrogate for assessing the long-term efficacy of the ablation lesion set itself, as it is heavily confounded by these transient perioperative factors. This perspective, however, stands in contrast to a substantial body of evidence suggesting that EPOAF is an independent risk factor for increased early and long-term mortality, as well as thromboembolic events such as stroke (15, 16). In minimally invasive cardiac surgery, prolonged cardiopulmonary bypass and aortic cross-clamp times are often observed. Increased myocardial ischemia time exacerbates oxidative stress, which may exert a proarrhythmic effect in the postoperative period. However, following propensity score matching, the incidence of early postoperative atrial fibrillation (POAF) was comparable between the minimally invasive and conventional sternotomy groups, recorded in 20 (33%) and 72 (38%) patients, respectively (*p* = 0.80). Furthermore, based on unpublished data from our cohort, we found no association between early POAF and long-term cardiovascular events.

The present study has several important limitations. First and foremost, it should be noted that this was a single-center retrospective study. This design inherently limits the ability to fully account for the influence of unmeasured confounding factors, despite the application of advanced statistical methods. A key consideration is the inherent year-related bias typical of most retrospective studies. The primary strength of the current study in this regard is that patients in both groups remained equally balanced over the entire study period (Supplementary Table S1). Another significant limitation is the progressive attrition of follow-up data over time, which may have compromised the accuracy of the long-term efficacy assessment. Furthermore, despite a generally similar set of ablation lines, the final choice of the specific lesion set was left to the surgeon's preference. This variability in the surgical approach represents a potential source of bias and could have influenced the outcomes of atrial fibrillation treatment.

## 5 Conclusion

The minimally invasive approach for combined mitral valve surgery and cryoablation for atrial fibrillation represents a technically more demanding procedure, as evidenced by longer CPB and ACC times. Nonetheless, this approach demonstrates not only overall survival comparable to conventional sternotomy but also comparable rates of major adverse cardiac and cerebrovascular events, freedom from recurrent atrial arrhythmias, and the need for permanent pacemaker implantation. It is important to emphasize, however, that the choice of surgical approach must always be guided by the surgical team's expertise, the patient's baseline characteristics, and individual risk assessment.

## Data Availability

The original contributions presented in the study are included in the article/Supplementary Material, further inquiries can be directed to the corresponding author.
