# Supplementary material for: Minimally invasive or conventional sternotomy for concomitant mitral valve surgery and surgical ablation for atrial fibrillation: propensity score study
Source: Front Cardiovasc Med. 2026 Jul 28;13:1821864. doi: 10.3389/fcvm.2026.1821864 (PMC13457103; doi:10.3389/fcvm.2026.1821864)
Supplement: Supplementary file 1 [file Datasheet1.docx]

**Supplementary Figure 1** Arrhythmias requiring hospital admission for the unmatched (A) and matched (B) cohorts. CS - conventional sternotomy group, HR – hazard ratio, MICS - minimally invasive cardiac surgery group.

A

B

**Supplementary Figure 2** Permanent pacemaker implantation after discharge for the unmatched (A) and matched (B) cohorts. CS - conventional sternotomy group, HR – hazard ratio, MICS - minimally invasive cardiac surgery group.

A

B

**Supplementary Table 1 Number of operations per year in each group**

| Year | 2012 | 2013 | 2014 | 2015 | 2016 | 2017 | 2018 | 2019 | 2020 | 2021 | 2022 | 2023 | 2024 |
| --- | --- | --- | --- | --- | --- | --- | --- | --- | --- | --- | --- | --- | --- |
| Group |  |  |  |  |  |  |  |  |  |  |  |  |  |
| MICS | 2 | 6 | 4 | 5 | 9 | 2 | 5 | 3 | 8 | 4 | 4 | 5 | 4 |
| CS | 8 | 14 | 12 | 10 | 16 | 18 | 25 | 19 | 12 | 15 | 14 | 13 | 15 |

**Abbreviations:** CS - conventional sternotomy group, HR – hazard ratio, MICS - minimally invasive cardiac surgery group.
